# Decentralized Sensor Fusion for Ubiquitous Networking Robotics in Urban Areas

**DOI:** 10.3390/s100302274

**Published:** 2010-03-19

**Authors:** Alberto Sanfeliu, Juan Andrade-Cetto, Marco Barbosa, Richard Bowden, Jesús Capitán, Andreu Corominas, Andrew Gilbert, John Illingworth, Luis Merino, Josep M. Mirats, Plínio Moreno, Aníbal Ollero, João Sequeira, Matthijs T.J. Spaan

**Affiliations:** 1 Institut de Robòtica i Informàtica Industrial, CSIC-UPC, Barcelona, Spain; E-Mails: cetto@iri.upc.edu (J.A.-C.); acoromin@iri.upc.edu (A.C.); jmirats@iri.upc.edu (J.M.M.); 2 Instituto Superior Técnico & Institute for Systems and Robotics, Lisbon, Portugal; E-Mails: mafb@isr.ist.utl.pt (M.B.); plinio@irs.ist.utl.pt (P.M.); jseq@isr.ist.utl.pt (J.S.); mtjspaan@isr.ist.utl.pt (M.T.J.S.); 3 Centre for Vision Speech and Signal Processing, University of Surrey, Guildford, UK; E-Mails: r.bowden@surrey.ac.uk (R.B.); a.gilbert@surrey.ac.uk (A.G.); j.illingworth@surrey.ac.uk (J.I.); 4 Pablo de Olavide University, Seville, Spain; E-Mail: lmercab@upo.es; 5 Robotics, Vision and Control Group, University of Seville, Seville, Spain; E-Mail: aollero@cartuja.us.es; 6 Center for Advanced Aerospace Technology, Seville, Spain

**Keywords:** network robot systems, distributed sensors, robot sensors, camera network

## Abstract

In this article we explain the architecture for the environment and sensors that has been built for the European project URUS (Ubiquitous Networking Robotics in Urban Sites), a project whose objective is to develop an adaptable network robot architecture for cooperation between network robots and human beings and/or the environment in urban areas. The project goal is to deploy a team of robots in an urban area to give a set of services to a user community. This paper addresses the sensor architecture devised for URUS and the type of robots and sensors used, including environment sensors and sensors onboard the robots. Furthermore, we also explain how sensor fusion takes place to achieve urban outdoor execution of robotic services. Finally some results of the project related to the sensor network are highlighted.

## 1. Introduction

In the URUS project [1] we needed to interconnect and manage multiple services that enable urban robots to perform social urban tasks. To this end, we have designed an architecture that takes advantage of all information available within a large set of ubiquitous sensors, including fixed cameras, wireless sensors, Mica2 sensors and various sensing devices onboard the robots, such as cameras and laser range finders. Two characteristics of this architecture are of particular relevance: it is a highly distributed architecture, and it is scalable. Contrary to other architectures for multiple robot interoperability, our system does not make use of a central server that receives and combines all the information available to obtain, for instance, a fused estimation of a given variable, say a tracked person position [2]. Such kind of systems are dependent on a central node, thus not robust to communication failures, latencies or drop outs, and they do not scale well with the number of nodes. In the URUS architecture, we opted for a decentralized system in which each subsystem only manages local information and exchanges with its peers local estimates of any given variable. Decentralized data fusion takes place by means of an information filter (IF), dual to the more common Kalman filter typically used for data fusion [3]. The IF has very nice characteristics for decentralization, with applications in other robotics contexts such as aerial robot decentralized perception [4]. One of the applications of decentralized data fusion in URUS is person tracking and guiding, employing the local sensors of the robot and the environment camera network, efficiently coping with occlusions, and single module tracking failures.

The article is organized as follows. We first present the objectives of the URUS project, the partners and the robot sites where the experiments take place. Then, we explain the distributed URUS architecture and the sensors in a network that combines cameras and Mica2 sensors. Next, we describe the sensors that are included in some of the robots used for the experiments. The robotic systems described in this paper include Tibi and Dabo from the Institut de Robòtica i Informàtica Industrial (CSIC-UPC), Romeo from the Asociación de Investigación y Cooperación Industrial de Andalucía (AICIA) and a fleet of Pioneer robots from the Instituto Superior Técnico (IST). Next we describe how information fusion from several sensors takes place, and the software architecture that we have developed to manage such heterogeneous set of sensors and systems. Finally, we explain how we use these sensors for several robot services as required by some of the URUS experiments, including for instance robot localization and people tracking.

## 2. The URUS Project

### 2.1. Objectives of the URUS Project

The general objective of the URUS project is the development of new ways for the cooperation between network robots and human beings and/or the environment in urban areas, in order to achieve efficiently tasks that for single systems would be too complex, time consuming or costly. For instance, the cooperation between robots and video cameras can solve surveillance problems for blind spots in urban areas, or the cooperation between robots and wireless communications devices can improve efficiency in people assistance services. The focus of the project is in urban pedestrian areas, for which there exists a growing interest in reducing the number of cars and improving the quality of life. Network robots become an important instrument towards these goals.

Network robots is a new concept that integrates robots, sensors, communications and mobile devices in a cooperative way. Meaning that, not only a physical connection between these elements exists, but also, that there is a need for the development of novel intelligent cooperation methods for task oriented purposes, new ways of communication between the different elements, and new robot mobility methods using the ubiquity of sensors and robots.

The URUS project is focused on designing and developing a network of robots that in a cooperative way interact with human beings and the environment for tasks of guidance and assistance, transportation of goods, and surveillance in urban areas. Specifically, our objective has been the design and development of a networked robot architecture that integrates cooperating urban robots, intelligent sensors (video cameras, acoustic sensors, *etc*.), intelligent devices (PDA, mobile telephones, *etc*.) and communications. The main scientific and technological challenges that have been addressed during the course of the project are: navigation and motion coordination among robots; cooperative environment perception; cooperative map building and updating; task negotiation within cooperative systems; human robot interaction; and wireless communication strategies between users (through mobile phones, PDAs), the environment (cameras, acoustic sensors, *etc*.), and the robots.

**Figure 1.**
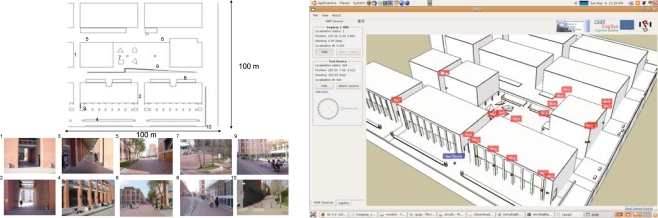
Barcelona Robot Lab.

Moreover, we have devised two demonstration scenarios in the Barcelona Robot Lab, a 10,000m^2^ area devoted to urban robotics experimentation (Figure 1). Scenario 1 involves transporting a person or goods to a destination; and Scenario 2 is devoted to drive people slowly and orderly towards the main exit in public spaces at closing hour. In the first case, a person calls, by means of a mobile phone, for a robots in order to receive the service. The robot that has the transport functionality, is available and closest to that person, approaches the person, identifies the person, and guides him or her to the requested final destination. All this, with the aid of the distributed sensor network for the tasks of localization, identification, guidance, and robot navigation. In the second case, the trigger signal for the surveillance service of public space is the closing time or a human gesture. Then, the appropriate robots available for this service approach the area where people is gathered and directs them to leave the space.

### 2.2. Project Participants

The project participants are:

- Institut de Robòtica i Informàtica Industrial, CSIC-UPC, Barcelona, Spain
- Laboratoire d’Analyse et d’Architecture des Systèmes, CNRS, Toulouse, France
- Swiss Federal Institute of Technology Zurich, Switzerland
- Asociación de Investigación y Cooperación Industrial de Andalucía, Seville, Spain
- Scuola Superiore di Studi Universitari e di Perfezionamento Sant’Anna, Pisa, Italy
- Universidad de Zaragoza, Spain
- Institute for Systems and Robotics, Instituto Superior Técnico, Lisbon, Portugal
- University of Surrey, Guildford, UK
- Urban Ecology Agency of Barcelona, Spain
- Telefónica I + D, Spain
- RoboTech, Italy

### 2.3. Barcelona Robot Lab

**Figure 2.**
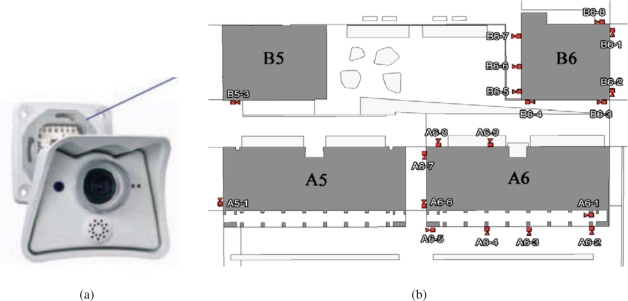
Camera network. (a) Colour video camera deployed in the URUS project. (b) The position of the cameras within the Barcelona Robot Lab.

The Barcelona Robot Lab, built within the context of this project, is an outdoor urban experimental robotics site located at the Universitat Politècnica de Catalunya (UPC) campus, which includes 6 university buildings over a 10,000 m^2^ area. In this area we have placed 21 fixed color video cameras connected through a Gigabit Ethernet connection to a computer rack. The position of the cameras is shown in Figure 2, with varying density in the positioning of the cameras, according to the various objectives of the project. Specifically, there are five cameras directed towards the Computer Science School (FIB) square, to have enough resolution for the detection of human gestures. There is also increased density in the positioning of the cameras in front of building A6, for the same reason. The rest of the area is covered with sparser density only to guarantee almost complete coverage of the area during people and robot tracking.

This outdoor facility is also equipped with 9 WLAN antennas with complete area coverage. Specifically for this project, we have installed the IEEE 802.11a protocol, contrary to the more common b/g/ or n networks. The reason was to limit as much as possible interferences with the university WLAN also covering the same area. The laboratory is also equipped with 802.15.4 wireless sensors for location purposes. Further information on the Barcelona Robot Lab experimental site can be seen at (http://www.urus.upc.es).

### 2.4. ISRobotNet

**Figure 3.**
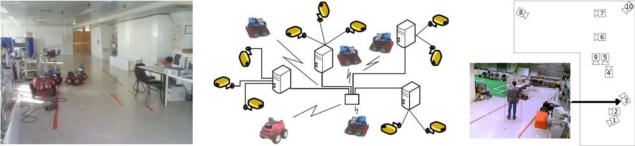
ISRobotNet.

The Institute for Systems and Robotics at Instituto Superior Técnico, in Lisbon, has developed also a testbed for NRS, the Intelligent Sensor and Robot Network (ISRobotNet), that enables testing a wide range of perception and robot navigation techniques, as well as human robot-interaction, distributed decision making, and task and resource allocation. The ISRobotNet testbed, also part of the URUS project, is composed of a 160 m^2^ indoor area with 10 webcams placed at the ceiling such that some of their fields of view do not overlap. The cameras are distributed in 4 groups, each of which is managed by its own computer for image acquisition. The managing computers are connected to the global ISR/IST network and can be accessed by duly authorized external agents. Ongoing work will extend the number of cameras and the usable indoor space to include multiple floors. Robots will use the same elevators as ordinary people to move between floors. Besides the camera sensors, four Pioneer ATs and one ATRV-Jr robots are available. Each of the robots is equipped with sonar, onboard cameras, laser range finder and is Wi-Fi connected to the network. Figure 3 shows one of the floors at ISR/IST where the testbed is implemented and a map with the camera fields of view.

ISRobotNet is built around the kernel of a service-oriented architecture (MeRMaID) [5], and extended to use the YARP networking software [6]. The testbed is being used to develop different components, namely, cooperative perception, people and robot detection and tracking using the camera network, and decision making. Arbitrary computational resources can be distributed over the network for fully decentralized use. Basic services such as robot teleoperation and direct image acquisition and recording from the camera network, as well as event logging, are also available.

## 3. The URUS Architecture

**Figure 4.**
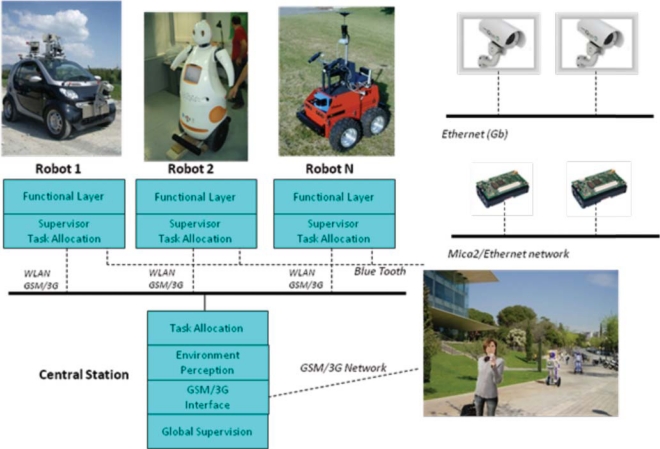
URUS global architecture.

The sensor architecture of URUS has being designed to manage distributed information coming from different sensors and systems, and they have to be used for flexible and dynamic robot services. The architecture is distributed among subsystems which are interconnected through various means, Ethernet cable, WLAN and GSM/3G (see Figure 4).

The architecture is divided in three layers:

- Environment Layer:
  – The networked cameras oversee the environment and are connected through a Gigabit connection to a rack of servers.
  – The wireless Zigbee sensors all communicate to a single subsystem which is also connected to the system through one computer.
  – The WLAN environment antennas are connected through the Gigabit connection to the rack servers.
  – People use devices to connect to the robots and the environment sensors. For instance, a mobile phone with PDA features is connected through GSM/3G to the system.
- Robot Sensor Layer:
  – The robots have their own sensors connected through proprietary networks (usually Ethernet) which are connected through WLAN and GSM/3G to the system. A proprietary communications service has been developed to transparently switch between WLAN and 3G depending on network availability.
- Server Layer:
  – The server rack (8 servers with 4 cores each) are connected through Ethernet to the Environment Layer and the Robot Sensor Layer.

The network of cameras is used to detect, track and identify people gestures in the urban area, as well as to detect and identify the robots. They can also be used for other services, such as surveillance, obstacle detection, *etc*. The wireless sensors, in this case Mica 2 sensor motes, are used mainly to enhance person and robot localization, based on radio signals. The Mica2 localization estimates are robustly fused with map-based localization methods in a decentralized way. Robot localization using GPS signals is not possible in the Barcelona Robot Lab, as in many urban areas due to satellite occlusion. The localization of the robots is so important, that we need to use as many methods as possible in order to minimize localization uncertainty.

The sensors onboard the robots are used mainly for navigation, localization and security; for robot and person identification, including the identification of human gestures and actions; and for human robot interaction.

Robots share information with other robots and with the server layer, through WLAN and GSM/3G. If WLAN is lost, for example when a robot crosses an area where the signal strength of the assigned AP (Access Point) is lost, then the robot either connects to another AP, or the robot connects to the system through GSM/3G.

The fully distributed software architecture is developed over YARP [6]. It can be used on any type of robots, sensors and human wearable devices. Specifically, we have developed standard procedures for connecting software modules allowing the share of information among the robots, sensors and human wearable devices.

At the level of sensor fusion for estimating system variables such as robot or people localization, data was fused in a decentralized form, with each component implementation independent from each other. However, at the experiment level, a central station was devised to handle task allocation and human operator commands through a GSM interface. This station had a finite state automata that took care of processing the experiment scripts and handling contingencies.

## 4. Sensors in the Urban Site

### 4.1. Camera Network

The camera network consists of 21 IP color video cameras distributed around the Barcelona Robot Lab. The cameras are connected to each building switch with a Gigabit link. From each building, there is a Gigabit connection to the computer rack with servers dedicated to process URUS software. These servers are in a rack located in another building 200m away from the NRS (Network Robot System) area. Each camera has a dedicated IP and is only accessible from the proprietary network that we have built. The parameters of the cameras can be modified independently and they include a time stamp that is used in the tracking functions.

The camera network serves as a mean to detect, localize and map environmental information in a globally coherent frame of reference. Persons and robots must be localized in a unique coordinate system even though they are observed by distant cameras. We perform image-feature registration for camera calibration. In contrast to other approaches that use a calibration pattern or stereo geometry over architectural features to infer depth, depth information is obtained from the range map. The reason to use the range map to geometrically relate the cameras is because no overlapping fields of view are available in the camera network. Additionally, being an outdoor system, it is constantly susceptible to weather conditions, such as rain and wind, and thus it is expected to have slight but visible positioning and orientation changes from time to time. The calibration methodology must therefore encompass simple self-adjusting mechanisms.

The development of powerful laser sensors combined with Simultaneous Location and Mapping (SLAM) methodologies [7, 8] allow the possibility to have available high precision Laser Range Finder (LRF) data registered over large areas. These large outdoor LRF datasets have started recently to be acquired also for the purpose of creating robot navigation systems in urban areas. The LRF map is acquired over the complete area of the network and, in particular, contains the areas corresponding to the fields of view of the cameras. We exploit these novel technologies proposing a methodology for calibrating an outdoor distributed camera network using LRF data.

**Figure 5.**
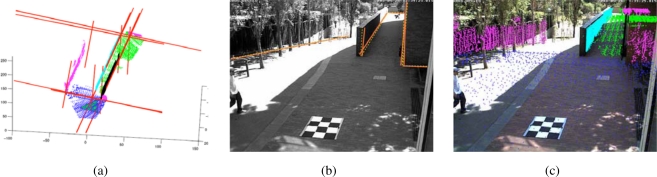
Camera calibration. (a) Lines are automatically extracted by intersecting planes on the LRF map, (b) The optimization method registers these 3D features with 2D lines on the images, and (c) Final reprojection of the segmented laser data after calibration.

The laser based map, whose construction is discussed further in this section, is used as external information to aid the calibration procedure of the distributed camera network. For our case, in which the cameras have non-overlapping fields of view, it is impossible to estimate the relative position between them unless external data is used to refer the camera calibration parameters to a global reference frame. Since calibration inevitably requires some user intervention, in large camera networks this can be a very tedious procedure if one does not develop practical and semiautomated methods that facilitate and speed up user input. See Figure 5.

The idea of the approach is the following: in a first stage, the LRF map is registered to an aerial view of the site and the user sets up the position and nominal calibration parameters of the cameras in the network. This allows user selection of an initial camera field of view onto the LRF area of interest likely to be observed by each camera. In a second stage, lines extracted from the LRF area of interest are represented in the nominally calibrated camera coordinate system and are reprojected to the real-time cameras’ acquired images. This allows the user to perceive the calibration errors and input information to a non-linear optimization procedure that refines both intrinsic and extrinsic calibration parameters. The optimization process matches 3D lines to image lines. The 3D lines are extracted by intersecting planes on the segmented LRF set. A novel approach to 3D range segmentation based on local variation is used [9]. To show the applicability of the calibration results, homographies of the walking areas are computed [10].

### 4.2. Mica2 Network

Latest advances in low-power electronics and wireless communication systems have made possible a new generation of devices able to sense environmental variables and process this information. Moreover, they have wireless communication capabilities and are able to form ad-hoc networks and relay the information they gather to a gateway. This kind of system is usually called a Wireless Sensor Network (WSN).

**Figure 6.**
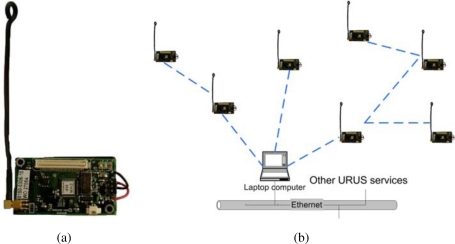
Mica2 network. (a) One of the sensor nodes employed in the system. (b) Scheme of the integration of the nodes into the architecture.

In URUS, a network of wireless Crossbow’s Mica2 sensor nodes is deployed in the urban scenario. Each Mica2 node (see Figure 6, a) has a suite of sensorial devices, including accelerometers, light and sound sensors, humidity sensors, *etc*., a tiny processor, based on the ATmega128L microcontroller, and a communication module on the 868/916 MHz band. These sensor nodes can be used for monitoring applications. In addition, the signal strength received by the set of static nodes of the WSN (Received Signal Strength Indicator, RSSI) can be used to infer the position of a mobile object or a person carrying one of the nodes. And thus, one of the applications developed in the project is an algorithm for mobile sensor tracking, which can be used for tracking persons in urban scenarios.

These sensors are integrated within the URUS system through a gateway which receives the information from the sensor nodes (see Figure 6b). The sensor nodes build themselves an ad-hoc network to send all the information to this gateway. In the gateway, a service that provides estimations on the position of mobile nodes is placed.

### 4.3. Site Map

We need to build the required maps for our heterogeneous fleet of service robots in urban settings [1]. With these maps the robots should be able to perform path planning and navigate to accomplish their tasks, such as guidance, assistance, transportation of goods, and surveillance. The experimental area has several levels and underpasses, poor GPS coverage, moderate vegetation, several points with aliasing (different points in the environment which cannot be disambiguated form local sensory data), large amounts of regularity from building structures, and sunlight exposure severely subject to shadows.

**Figure 7.**
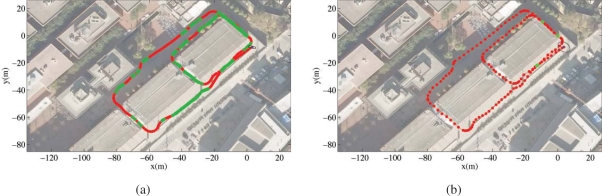
Filtered trajectory (in red) using encoder and visual odometry on a dataset collected at the Barcelona Robot Lab. Loop closure links are displayed in green, the black ellipse indicates the final robot location and its associated covariance at a 95% confidence level. (a) standard approach: incorporating all poses and all links to the filter; and (b) proposed approach: incorporating only relevant poses and links.

Our technique to Simultaneous Localization and Mapping (SLAM) is described in detail in [7, 11, 12], and is summarized here. We introduce a principled on-line approach for Pose SLAM, the variant of SLAM in which the state vector contains a history of visited robot locations, which only keeps non-redundant poses and highly informative links (see Figure 7). This is achieved by computing two measures; the distance between a given pair of poses and the mutual information gain when linking two poses. In [11], we show that, in Pose SLAM, the exact form of these two measures can be computed in constant time. When compared to other existing approaches [13], the proposed system produces a more compact map that translates into a significant reduction of the computational cost and a delay of the filter inconsistency, maintaining the quality of the estimation for longer mapping sequences.

By applying the proposed strategy, the robot closes only few loops and operates in open loop for long periods, which is feasible using recent odometric techniques [14, 15]. Our Pose SLAM approach includes a novel state recovery procedure at loop closure that takes advantage of the inherent sparsity of the information matrix scaling linearly both in time and memory. This computational cost is further amortized over the period where the robot operates in open loop, for which we introduce a factorization of the cross- covariance that allows the state to be updated in constant time. Thus, the proposed state recovery strategy outperforms state of the art approaches that take linear time for very sparse matrices (*i.e.*, when the robot operates in open loop), but are worst case quadratic when many loops are closed [13]. After this, the bottleneck for real time execution is not state recovery, but data association. That is, detecting poses close to the current one for which feature matching is likely. Exploiting the factorization of the cross-covariance, we introduced also in [11] a tree-based method for data association using interval arithmetic to encode the internal nodes of the tree. The main advantage of that method is that it improves the search up to logarithmic time. Moreover, by taking into account the cross-covariances from the very beginning it also avoids false positives, typically present in existing tree-based data association techniques[16]. This offers the possibility to use Pose SLAM for mapping large scale environments, such as the Barcelona Robot Lab.

**Figure 8.**
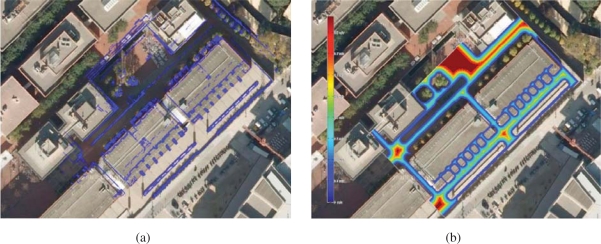
Traversability map built from 2D layers of three-dimensional aligned point clouds. (a) 2D layer superimposed on an aerial image. (b) Corresponding traversability map. Velocity varies from 0 m/s (blue) to 1 m/s (red).

**Figure 9.**
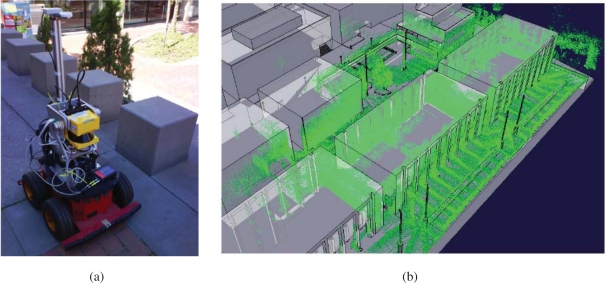
Site map. (a) Custom built 3D laser range scanner mounted on a Pioneer robotic platform. (b) Top view of the 3D map.

Aerial views of the site are shown in Figures 7 and 8. In order to build the maps described in this paper, we built our proprietary 3D scanning system, using a Leuze RS4 scanner and controlling its pitch with a DC motor and a computer. The system was installed atop an Activmedia Pioneer 2AT robotic platform. The system yields 3D point clouds with ranges up to 30 m, and sizes of about 76,000 points. The sensor noise level is 5 cm in depth estimation for each laser beam. Figure 9 portrays the complete device. The robot was tele-operated through the site along a path of over 600m (see Figure 9b).

The figure contains results from state augmentation by concatenation of ICP computed motion constraints through the EIF Pose SLAM algorithm that closes 19 loops in the trajectory [7, 11, 12]. The figure also shows a comparison of the mapping results to a manually built CAD model of the experimental site. This model is made using geo-referenced information.

## 5. Sensors Included in Urban Robots

### 5.1. Sensors in the Robots Tibi and Dabo - Architecture and Functionalities

**Figure 10.**
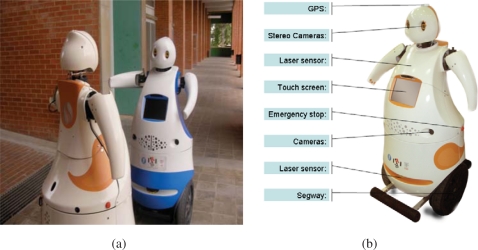
(a) Tibi and Dabo; (b) Tibi sensors.

Tibi and Dabo (Figure 10) are two robots built at IRI with functionalities to navigate in urban areas, to assist and guide people from one origin to a destination. Traction is based on Segway RMP200 robotic platforms, and they have been customized with a variety of sensors:

- Sensors for navigation: Two Leuze RS4 and one Hokuyo laser rangefinders, as well as Segway’s own odometric sensors (encoders and IMU).
  – The first Leuze rangefinder is located in the bottom front with a 180° horizontal view. This sensor is used for localization, security and navigation.
  – The second Leuze rangefinder is located in the bottom back, also with a 180° horizontal view. This sensor is used for localization, security and navigation.
  – The Hokuyo rangefinder is located in the front, but placed vertically about the robot chest, also with a 180° field of view. It is used for navigation and security.
- Sensors for global localization: GPS and compass.
  – The GPS is used for low resolution global localization, and can only be used in open areas where several satellites are visible. In particular, for the URUS scenario, this type of sensor has very limited functionality due to loss of line of sight to satellites from building structures.
  – The compass is used also used for recovering robot orientation. Also, in the URUS scenario, this sensor has proven of limited functionality due to its large uncertainty in the presence of metallic structures.
- Sensors for map building: One custom built 3D range scanner and two cameras.
  – The two cameras are located to the sides of the robots and facing front to ensure a good baseline for stereo triangulation, and they are used for map building in conjunction with the laser sensors. These cameras can also be used for localization and navigation.
  – A custom built 3D laser range finder unit has been developed in the context of the project. This unit, placed on top of a Pioneer platform has been used to register finely detailed three dimensional maps of the Barcelona Robot Lab that allow localization, map building, traversability computation, and calibration of the camera sensor network.
  – Vision sensors: One Bumblebee camera sensor.
  – The Bumblebee camera sensor is a stereo-vision system that is used for detection, tracking and identification of robots and human beings. Moreover we use this camera as image supplier for robot teleoperation.
- Tactile display:
  – The tactile display is used for Human Robot Interaction (HRI), to assist people and to display information about the status of the robot, as well as task specific information, such as destination information during a guidance service.

**Figure 11.**
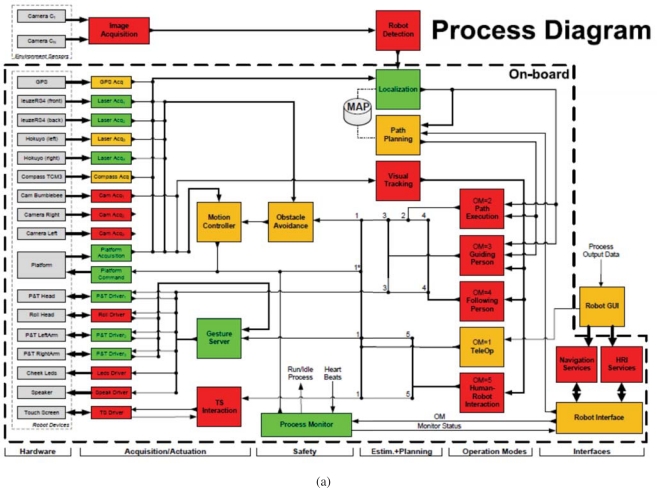
Internal architecture of Tibi & Dabo.

The internal architecture of these robots can be seen in Figure 11. We can see all hardware and sensors on the left side and their connection to the Acquisition/Actuation system. Colors in the figure indicate development state at an early stage of the project. Green indicates software modules that were completed, yellow indicates software modules that were in development at that time, and red indicates software modules that were still not developed. The second Acquisition/Actuation subsystem, second column in the figure, includes all drivers that connect hardware and sensors to the localization, motion controller, visual tracking, obstacle avoidance, gesture server and TS interaction modules. The safety subsystem in the third column uses the process monitor to control the robot platform, using the information from the robot sensors and the heart beats of the robot systems. The Estimation and Planning subsystem use the localization and path planning to control the robot motion. The different operation modes for these robots are: path execution, guiding person, tele-operation and human robot interaction. Each one of these operation modes are used in the URUS project to give specific services.

### 5.2. Romeo Sensors—Architecture and Functionalities

**Figure 12.**
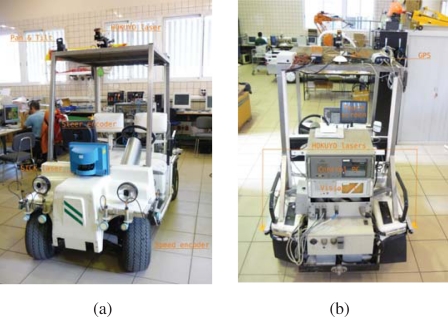
Sensors on board Romeo. Sensors for localization (GPS, gyro, encoders, Sick laser), map building and navigation (laser rangefinders). (a) front view; (b) rear view.

Romeo is an electric car modified with sensors and actuators to navigate autonomously in outdoor scenarios, including urban environments. Figure 12 shows the robot and its main sensors:

- Odometric sensors: Romeo has wheel encoders for velocity estimation, and a KVH Industries’ gyroscope and an Inertial Measurement Unit (IMU) for angular velocity estimation.
- Rangefinders: Romeo has one SICK’s LMS 220-30106 laser rangefinder located in the frontal part of the robot, at a height of 95 cm, for obstacle avoidance and localization. Moreover, it has 2 Hokuyo’s URG-04LX (low range, up to 4 meters) in the back for backwards perception, and 1 Hokuyo’s UTM-30LX (up to 30 meters) at the top of Romeo’s roof and tilted for 3D perception.
- Novatel’s OEM differential GPS receiver.
- Firewire color camera, which can be used for person tracking and guiding.
- Tactile screen, which is used for robot control and for human-robot interaction.

**Figure 13.**
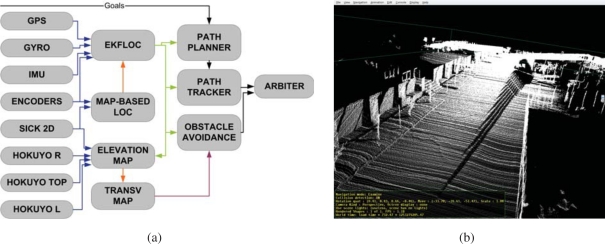
(a) basic architecture of Romeo. (b) 3D information obtained by Romeo from the Romeo lasers. Different obstacles can be identified, like stairs, trees or small steps.

Figure 13, a, shows the basic software architecture for robot navigation in pedestrian environments. For localization, Romeo fuses all its odometric sensors (encoders, gyroscope, IMU) with an Extended Kalman Filter (EKF-LOC) to estimate its 6D pose. In order to compensate the drift associated to odometry, Romeo carries a differential GPS receiver. However, as stated before, in urban environments, often the GPS measurements are not available, or are affected by multi-path effects, which can produce erroneous estimates, thus localization is map-based and only assisted by GPS. All robots, Tibi, Dabo, and Romeo use the same map-based localization mechanism developed by Corominas *et al.* [17, 18], and described further in this paper, allowing the cancelation of drift error at a rate of 1 Hz.

For navigation, Romeo combines the pose information from the localization algorithm with the data from its lasers to build a 3D representation of the environment (see Figure 13b). From this representation, it is possible to obtain a traversability map; that is, a grid in which each cell indicates if it can be drivable by Romeo or not, such as the one shown in Figure 8b. The analysis to decide if a place is drivable or not depends mainly on the slope at each point of the map. At the same time, mobile obstacles can be also identified. This is used by the obstacle avoidance module to command the robot.

**Figure 14.**
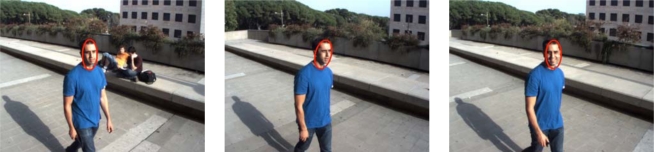
People tracking from Romeo for guidance.

Romeo carries on board cameras that can be used for person tracking and guiding. The algorithms employed for this are based on a combination of person detection and tracking. The tracking algorithm is based on the mean shift technique [19]. In parallel, a face detection algorithm is applied to the image [20], the results from the tracking and the detection applications are combined, so that the robot employs the face detector when the tracker is lost to recover the track. As a result, the robots can obtain estimates of the pose of a person face on the image plane (see Figure 14).

### 5.3. ISTRobotNet Architecture and Functionalities

**Figure 15.**
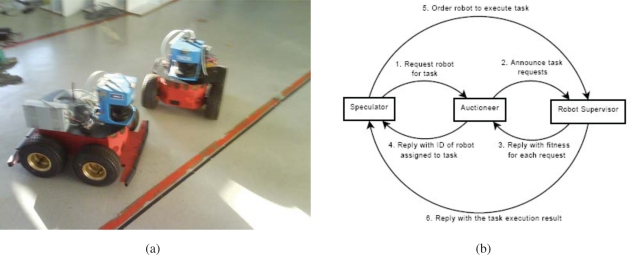
(a) IST robots and, (b) the auction based distributed approach to high level supervision.

IST is using Pioneer 3AT robots equipped with odometry, ultrasound, and laser range finder sensors (Figure 15). Basic navigation capabilities, namely path planning and obstacle avoidance, are available for each robot. Localization for these robots is also is based on the fusion of odometry and range information and the a priori defined world map. Contrary to the robots that navigate in the Barcelona Robot Lab, the CARMEN package is used for map building and localization at the ISRobotNet site. A grid is superimposed on the map obtained with CARMEN, and the centers of each cell constitute the set of waypoints used in the robot navigation.

An auction based high level supervision is implemented by a distributed component [21]. This approach is flexible enough to allow fast prototyping in behavior development. The robot tasks consist in the use of navigation behaviors to reach a given cell in the grid map. The robot supervisor is synthesized by formulating POMDP’s for each possible task [22]. Thus, the robots task fitness is the value function of the POMDP for each task. The benefit of using the POMDP approach is that it is possible to compare the fitness of unrelated tasks, such as navigation and the use of onboard sensors for tracking.

The camera detection events are sent to the speculators, see Figure 15, which process this information and determine if a robot should approach the cell where the detection occurred. Since in general the number of detection events and mobile robots is different, an auction protocol is implemented to compute the optimal task assignment solution given the robots individual fitness values.

Experiments integrating all the components required for the URUS project have been carried out at the ISRobotNet. A typical experiment can be identified with a rendezvous scenario where a service robot meets a person detected by the camera network. Each camera detecting persons and robots associates an uncertainty measure (covariance matrix) to each localization hypothesis in world coordinates. When targets are observed by more than one camera, a Bayesian data fusion process joins the information from the several cameras and provides a posteriori distribution of the data, assuming Gaussian uncertainties.

**Figure 16.**
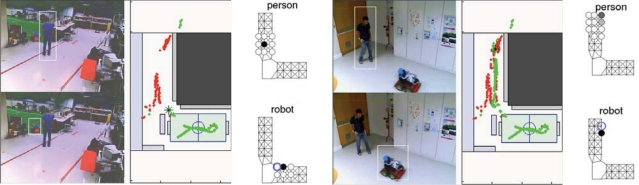
Rendezvous experiment. Integrated experiment at ISRobotNet.

Figure 16 shows (i) the detections provided by the camera network; (ii) the person and robot positions displayed in the 2D world map and (iii) the environment discrete grid cells. The maps show trajectories of persons and robots obtained by gathering information from all the cameras. False positive detections are due mainly to occlusions in the field of view of the cameras, but the false detection rate is very low and does not influence the probabilistic localization methods. The green and red dots indicate estimated robot and people trajectories, respectively. The box and circles map represents the likelihood of person and robot positions (the darker the cell, the higher the likelihood) and the blue circle shows the selected cell for commanding the next robot position, determined by the decision process. This information is then used to plan minimal cost paths accounting, for instance, for the relevance of visiting extra locations [23, 24].

## 6. Decentralized Sensor Fusion for Robotic Services

**Figure 17.**
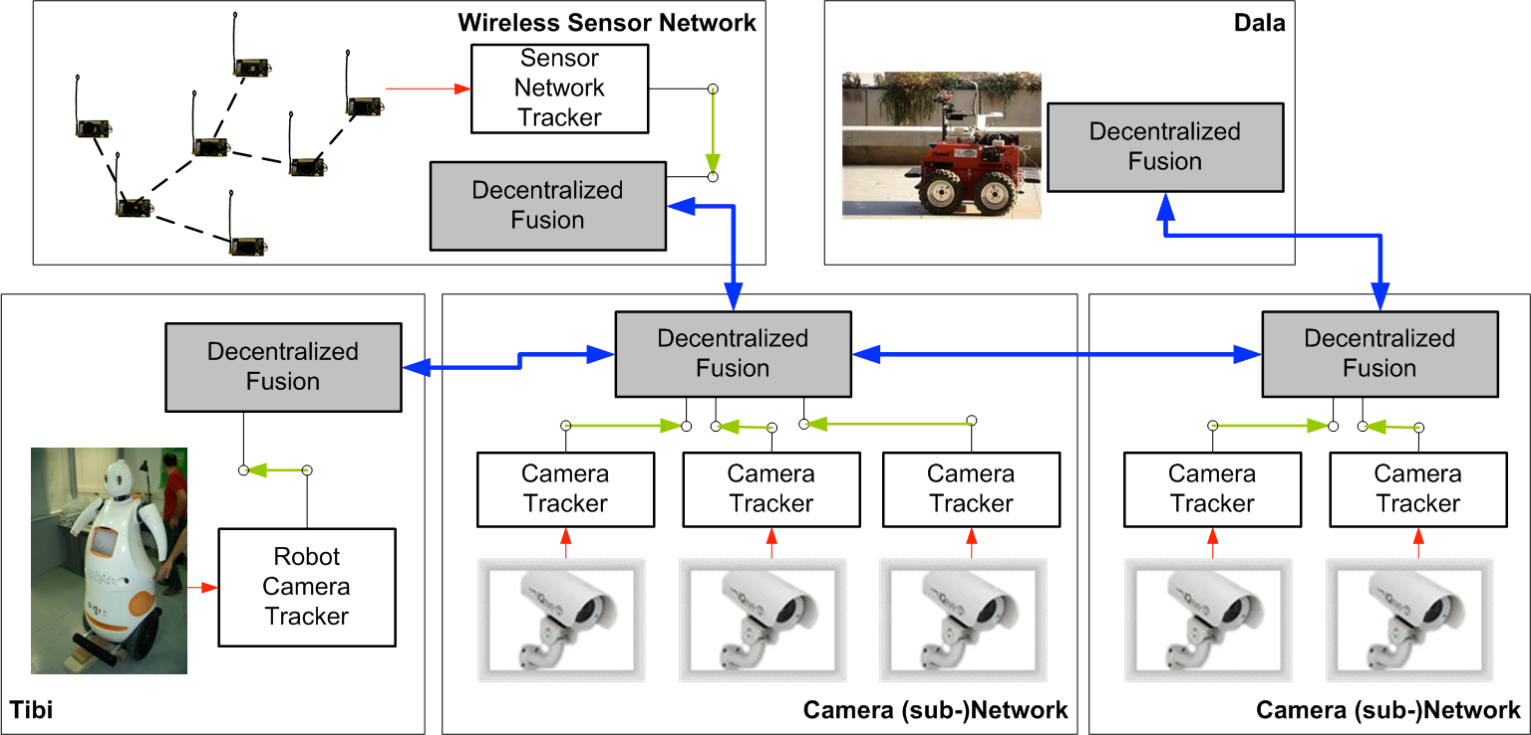
A block description of the sensor fusion architecture in URUS.

From the perception point of view, in the URUS system (Figure 17), the information obtained by the fixed camera network or the wireless sensor network is fused with the information each robot obtains about the environment to improve perception estimates. In a guiding task for instance, the targets identified by the camera network (see Figure 17, right) can be combined with the information from the other systems (robots, and WSN, see Figure 17) to improve the tracking of the person being guided. Combining information from several sources, allows to cope with occlusions, to obtain better tracking capabilities as information of different modalities is employed, and to obtain predictions for uncovered zones, as the robots can move to cover these zones.

One option is to have a centralized system that receives all the information and performs data fusion. However, such approach presents some drawbacks, as it does not scale well with the number of robots and other systems; the central element can be a bottleneck; and communication failures are critical. Therefore, in URUS we have implemented a decentralized estimation system (as shown in Figure 17). In a decentralized data fusion system [25, 26], there are a set of fusion nodes which only employ local information (data from local sensors; for instance, a camera subnet, or the sensors on board the robot) to obtain a local estimate on the quantity of interest (for instance, the position of the person being guided). Then, these nodes share their local estimates among themselves if they are within communication range. The main idea is that, as the nodes only use local communications, the system is scalable. Also, as each node accumulates information from its local sensors, temporal communication failures can be coped without losing information. Finally, fusing information from neighbor nodes allows for consistent global estimation in a decentralized form.

**Figure 18.**
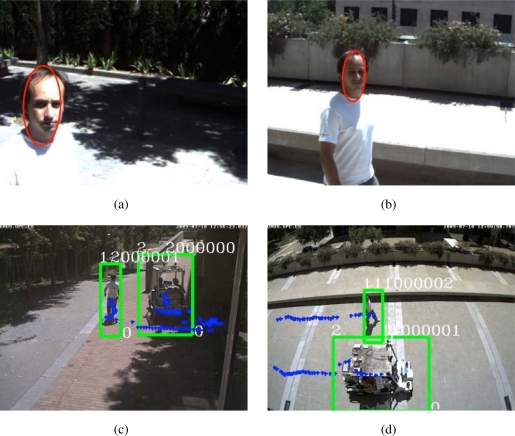
(a-b) Tracks obtained by the camera on board Romeo. (c-d) Tracks obtained by the camera network in the same experiment.

Using the trackers developed in the project, the camera network and the robots are able to obtain local estimates of the variable at hand (*i.e.*, the position of people on the image plane, see Figure 18). These estimates, characterized as Gaussian distributions (mean and covariance matrix) and the ones provided by the WSN, can be fused in order to obtain an accurate estimate of such variable. The Information Filter (IF), which corresponds to the dual implementation of the Kalman Filter (KF), is very suitable for decentralized data fusion using Gaussian estimates. While the KF represents the distribution using its first and second order moments (mean *μ* and covariance Σ), the IF employs the so-called canonical representation, encoded by the information vector *η* = Σ^−1^*μ* and the information matrix Ω = Σ^−1^. Prediction and updates for the linear IF are easily derived from the linear KF [3]. In the case of nonlinear motion models (*i.e.*, robot kinematics) and nonlinear measurement functions (*i.e.*, perspective projection on cameras), first order linearization leads to the Extended Information Filter (EIF).

The main interest of the EIF, compared to the EKF, is that it can be easily decentralized. In a decentralized approach, each node of the network employs only local measurements **z***_i_*, of the event at hand (as said, for instance, the position of the person as observed by a local sensor), to obtain a local estimate of such event (the person trajectory), represented by *η_i_* and Ω*_i_*, and then, shares this estimate with its neighbors. The information coming from neighboring nodes is locally fused in order to improve such local estimate. The decentralized fusion rule produces locally, the same result that would be obtained using a centralized EIF. This is because for the EIF, data fusion is an additive process [26]:

$$\begin{array}{c} \eta_{i}\leftarrow \eta_{i}+\eta_{j}-\eta_{\mathrm{ij}}\\ \Omega_{i}\leftarrow \Omega_{i}+\Omega_{j}-\Omega_{\mathrm{ij}} \end{array}$$

The update rules state that each node should sum up the information received from other nodes. The additional terms *η_ij_* and *Ω_ij_* represent the common information between nodes. This common information is due to previous communications between nodes, and should be removed to avoid double counting of information, known as rumor propagation. This common information is maintained by a separated EIF called channel filter [27]. This common information is locally estimated assuming a tree-shaped network topology, i.e., there exist no cycles or duplicated paths of information.

The decentralized system has as advantages that the system is scalable, as each fusion node employs only local communications. Moreover, communications dropouts do not compromise the system (although the performance can be degraded during the dropout). Another advantage of using delayed states is that the belief states can be received asynchronously. Each node in the network can accumulate evidence, and send it whenever it is possible. However, as the dimension of the state trajectories grow with time, the size of the message needed to communicate the estimate of a node also does. For the normal operation of the system, only the state trajectory over a limited time interval is needed, so these trajectories can be bounded. Note that the trajectories should be longer than the maximum expected delay in the network in order not to miss any measurements information.

## 7. Software Architecture to Manage Sensors Networks

The ever-increasing level of autonomy demanded by networked robotic systems is naturally leading to an increase in the complexity in their components. In robotics parlance these components and strategies are known as middleware, following similar designations in computer engineering, referring to computer software that connects software components or applications. MeRMaID (Multiple-Robot Middleware for Intelligent Decision-making) is a robot programming framework, used in URUS, whose goal is to provide a simplified and systematic high-level behavior programming environment for multi-robot teams, supported on a middleware layer [5]. This middleware layer uses the Service concept as its basic building block.

**Figure 19.**
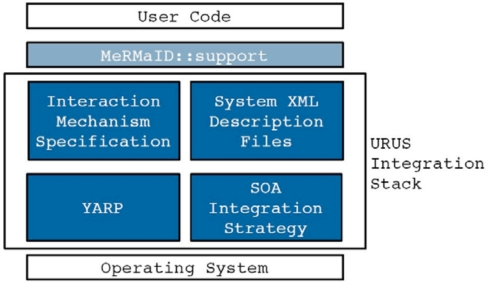
General structure overview of the middleware layer MeRMaID::support.

The middleware layer used in URUS extends that developed for RoboCup competitions [28]. The functional architecture, guides the development of the embedded components so as to improve overall dependability. This way, MeRMaID ensures that separately developed components will be more easily assembled together at integration time. The use of this architectural layer is not mandatory. The developer can build its application using only the support layer, without any constraints on how to connect subsystems. Figure 19 depicts an overview of the structure of both levels of MeRMaID. For a thorough description of MeRMaID and its functionalities, refer to [5].

Among the advantages in using the MeRMaID framework are (i) reduced time to have third part components fully integrated in to the system, and (ii) the automatic validation of the interactions among services, assuring the developer that all protocols and specifications are correctly followed. The integration of functionality provided by image processing software connected to the camera network in this setup is an example of quick integration with software not developed using MeRMaID. The software to detect people and robots was developed by an independent team without any constraint on how they would later integrate it with the rest of the system at ISRobotNet. Given that the software was able to detect events visible by the camera network that occur asynchronously, the Data Feed mechanism was chosen as appropriate to make this information available. In fact, the whole camera network can be seen as just a producer of a stream of asynchronous events. It was quite simple to insert a valid data feed mechanism in the camera network code. Developers only needed to write data to a YARP Port (which is YARP’s fundamental building block for communications) and populate the system’s description file with a declaration of what data they were exporting. This approach can be applied for any data producing software component in the system. Services developed using MeRMaID Support also benefit from simplified access and full syntactic validation of the sent data.

## 8. Some Results in the URUS Project

In this section we only explain briefly three examples developed in the URUS project, where a combination of sensors is used for specific robotic functionalities: localization using robot sensors and environment sensors; tracking people and detection of gestures; and tracking of persons and robots using Mica2 sensors.

### 8.1. Localization

One of the main functions that any robot operating in an urban environment must have is localization. Without this functionality the robots cannot perform missions like, “Go to destination X to pick up a person and guide him to destination Y”. The robot must first know its position and pose with respect to the global reference frame, and then know where is the X and Y positions are within that reference frame, to be able to navigate and guide the person. One could think this is a trivial task in an urban setting that could be solved for instance, with a GPS sensor. However, GPS signals are often unreliable for autonomous robot navigation in urban pedestrian areas: there is lack of satellite visibility for accurate position estimation. For this reason, we have developed a map-based position estimation method for precise and fast localization using the information sensed by the laser rangefinders installed in the robot, a GIS-augmented map of the environment, and the camera network. For the robots Tibi and Dabo, we use the front and rear laser sensors. Usually, the aggregation of evidence from laser data is sufficient for accurate map-based localization. Nonetheless, in some cases of aliasing, estimates on robot location coming from the camera network help disambiguate between similar localization hypotheses, and also as strong localization evidence in densely populated areas [17, 29]. The method in [30] is based on a particle filter approach [31], and is summarized here. Note that the localization mechanism described next accounts for a local node of the decentralized position estimation mechanism detailed in Section 6. Other nodes include for instance, each robot localization estimate coming from each of the cameras in the network.

Let $X_{r}^{t}=\left(x_{r}^{t},\;y_{r}^{t},\;\theta_{r}^{t}\right)$ be the robot state a time *t*, and assume it is limited to a bounding box in the three-dimensional pose space, $X_{r}^{t}\in \Gamma =\left\{\left(x_{\min},\;x_{\max}\right),\;\;\left(y_{\min},\;y_{\max}\right),\;\left(-\pi ,\;\pi \right)\right\}$. The particle filter steps are:

1. Propagation: All particles are propagated using the kinematic model of the robot and the odometric observation.
  $$X_{i}^{t}=f\;\left(X_{i}^{t-1},\;o_{0}^{t}\right);\;\forall i=1\ldots N_{p}$$
2. Correction: Particle weights are updated according to the likelihood of the particle state given the observations, *k* = 1. . . *N_B_*:
  $$w_{i}^{j}=w_{i}^{j-1}\;\prod_{k=1}^{N_{B}}\;p\left(X_{i}^{t}|o_{k}^{t}\right);\;\forall i=1\ldots N_{p}$$
  where $p\left(X_{i}^{t}|o_{k}^{t}\right)=L_{k}\;\left(o_{k}^{t},\;o_{k}^{g}\left(X_{i}^{t}\right)\right)$ and $L_{k}\;\left(o_{k}^{t},\;o_{k}^{g}\left(X_{i}^{t}\right)\right)$ is the likelihood function between two observations: the current one made by the *k*-th observer, $o_{k}^{t}$ (a ray traced from the laser scanner), and the expected one $o_{k}^{g}\left(X_{i}^{t}\right)$, computed using the *k*-th observation and the environment model. This $o_{k}^{g}\left(X_{i}^{t}\right)$ observation is called *synthetic* because is computed using models. We have as many likelihood functions as sensors we have, and they are included in the computation of this likelihood. For example, for the laser scan this function is:
  $$L_{1}\;\left(o_{1}^{t},\;o_{1}^{g}\;\left(x_{i}^{t}\right)\right)=\frac{1}{N_{L}}\sum_{j=1}^{N_{L}}\text{erfc}\frac{\sigma_{1,j}^{t}-\sigma_{1,i}^{g}\left(X_{i}^{t}\right)}{\sigma_{1}\sqrt{2}}$$
  where *σ*_1_ is the noise for the laser observation and
  $$\sigma_{1,i}^{g}\;\left(X_{i}^{t}\right)=\text{raytr}\;\left(x_{i}^{t},\;y_{i}^{t},\;\theta_{i}^{t}-\frac{\Delta \alpha }{2}+j\frac{\Delta \alpha }{N_{L}}\right)$$
  with Δ*α* the aperture angle of the laser scan and raytr(*X_p_*) is a function to evaluate the distance of a given position in the map, *X_p_*, with the closest obstacle of the map, following an extended ray casted at the proper heading. The details of the other two steps, set estimate and resampling, can be seen in [30].

#### Integration of Asynchronous Data

**Figure 20.**
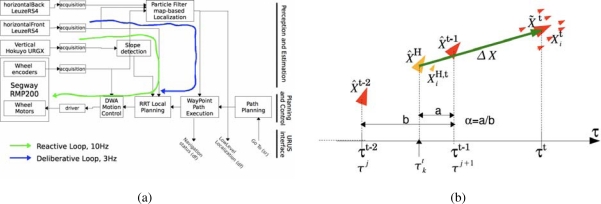
(a) Tibi and Dabo asynchronous navigation. (b) Particle propagation. Backward propagation of the particle $X_{i}^{t}$ when integrating observation $o_{k}^{t}$.

**Figure 21.**
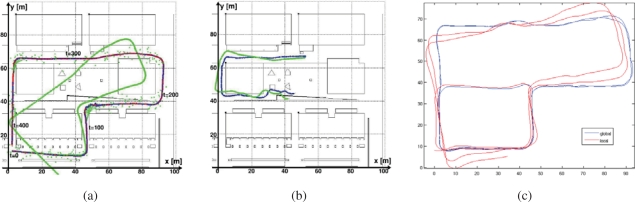
Robot localization. (a) Simulated results with multiple sensors. (b) Robot localization in the real outdoor campus environment with the robot Tibi. The red path indicates ground truth, the green path indicates robot odometry, and the blue path the resulting filter estimate. (c) Map based localization results in the same environment for the robot Romeo. The red path indicates local estimates, and the blue path indicates global localization.

Given the asynchronous nature of the data coming from the various sensors (laser scanners, odometry, and eventually GPS) (see Figure 20a), the particle filter integrates observations taking into account their time stamps. The filter does not propagate the particle set once per iteration as classical approaches do. Instead, the proposed algorithm propagates the particle set only when a new observation arrives with a time stamp greater than the last propagation. At this point, the filter propagates with the kinematic model and the odometric observation and, then, integrates the observation as a delayed one. In order to integrate delayed observations, the proposed approach maintains a delayed history of past estimates and back-propagates particles to compute observation models at those instances where particles were expected to be [32] (see Figure 20b). The experiments using this particle filter in the Barcelona Robot Lab can be seen in Figure 21.

### 8.2. Tracking People and Detecting Gestures Using the Camera Network of Barcelona Robot Lab

#### 8.2.1. The Method

The fixed cameras cover a wide area of the experiment site and are the basis for the fusion of the other sensors, they are able to track objects of interest both on and across different cameras without explicit calibration. The approach is based on a method proposed by Gilbert and Bowden [33]. Intra camera objects of interest are identified with a Gaussian mixture model [34] and are linked temporally with a Kalman filter to provide intra camera movement trajectories. When the object of interest enters a new camera field of view, the transfer of the object position estimate to the new camera is challenging since cameras have no overlapping fields of view, making many traditional image plane calibration techniques impossible. In addition, handling a large number of cameras means that traditional time consuming calibration is impractical. Therefore, the approach needs to learn the relationships between the cameras automatically. This is achieved by the way of two cues, modeling the color, and the inter-camera motion of objects. These two weak cues, are then combined to allow the technique to determine if objects have been previously tracked on another camera or are new object instances. The approach learns these camera relationships, and unlike previous work, it does not require a priori calibration or explicit training periods. Incrementally learning the cues over time allows for accuracy increase without supervised input.

#### 8.2.2. Forming Temporal Links between Cameras

**Figure 22.**
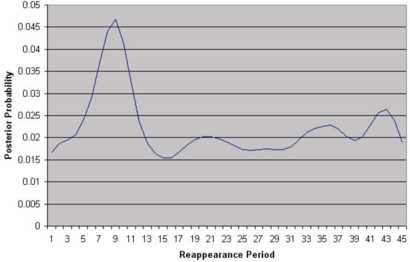
An example of a probability distribution showing a distinct link between two regions.

To learn the temporal links between cameras, we make use of the key assumption that, given time, objects (such as people) will follow similar routes inter-camera and that the repetition of the routes will form marked and consistent trends in the overall data. Initially the system is subdivided so that each camera is a single region. It identifies temporal reappearance links at the camera-to-camera level. After sufficient evidence has been accumulated, the noise floor level is measured for each link. If the maximum peak of the distribution is found to exceed the noise floor level, this indicates a possible correlation between the two blocks as shown in Figure 22. If a link is found between two regions, they are both subdivided to each create four new equal sized regions providing a higher level of detail. Regions with little or no data are removed to maintain scalability.

#### 8.2.3. Modelling Color Variations

The color quantization descriptor, used to form temporal reappearance links, assumes a similar color response between cameras. However this is seldom the case. Therefore, a color calibration of these cameras is proposed that can be learnt incrementally, simultaneously with the temporal relationships discussed in the section above. People tracked inter-camera are automatically used as the calibration objects, and a transformation matrix is formed incrementally to model the color changes between specific cameras.

The transformation matrices for the cameras are initialized as identity matrices assuming a uniform prior of color variation between cameras. When a person is tracked inter-camera and is identified as the same object, the difference between the two color descriptors, is modeled by a transform matrix. The matrix is calculated by computing the transformation that maps the person’s descriptor from the previous camera to the person’s current descriptor. This transformation is computed via SVD. The matrix is then averaged with the appropriate camera transformation matrix, and repeated with other tracked people to gradually build a color transformation between cameras.

#### 8.2.4. Calculating Posterior Appearance Distributions

With the weak cues learnt, when an object which leaves a region, we can model its reappearance probability over time as

$$P\left(O_{t}|O_{y}\right)=\sum_{\forall x}w_{x}P\left(O_{x,t}|O_{y}\right)$$

where the weight *w_x_* at time *t* is given as

$$w_{x}=\frac{\sum_{i=0}^{T}\;f_{\Phi }^{x|y}}{\sum_{\forall y}\;\sum_{i=0}^{T}\;f_{\Phi }^{x|y}}$$

representing the ratio of weak feature responses in region *x* given the feature has also been observed in region *y*. This probability is then used to weight the observation likelihood obtained through color similarity to obtain a posterior probability of a match. Tracking objects is then achieved by maximizing the posterior probability within a set time window.

In order to provide additional information of the objects of interest being tracked over the cameras, we also address the distinction of the objects by classifying them as a person or a robot.

#### 8.2.5. Classification of Objects of Interest as Person or Robot

The intra camera tracking algorithm provides a unique identifier for every object of interest based on color cues. In addition to the identifier, the discrimination of the objects between people and robots provides a useful cue for the fusion procedure. We propose to categorize the objects of interest as human or robots by the differences on their motion patterns, which are obtained from the optical flow [35]. We assume the robots are rigid bodies that produce flow vectors with very similar orientations, while a person produces patterns on different orientations.

The discrimination of the motion patterns relies on: (i) optical flow-based features and (ii) a learning algorithm with temporal consistency. We compare two types of flow features, which have been used previously to detect people: (i) Histogram of gradients (HOG) [36], which computes the histogram of the optic flow orientation weighted by its magnitude and (ii) Motion boundary histogram (MBH) [37], computed from the gradient of the optical flow. Similarly to HOG, this feature is obtained by the weighted histogram of the optical flow’s gradient.

In order to classify an object of interest at every frame, a boosting algorithm uses labeled samples of either HOG or MBH features in a binary problem: person *vs.* robot. We use the GentleBoost algorithm, which provides a framework to sequentially fit additive models (*i.e.*, weak learners) in order to build a final strong classifier. We select a weak learner that considers the temporal evolution of the features, the Temporal Stumps [35], which are an extension of the commonly used decision stumps. The output of the strong classifier at each frame is related to the person/robot likelihood.

#### 8.2.6. Gesture Detection

The key gesture to be recognized by the camera network is people waving. The importance of this specific gesture is due to its “universal” nature: it is used as an attention triggering and emergency indicator by most people independently of their culture. It is worth to point that state-of-the-art, high performance, hand gesture recognition systems still present robustness problems that limit their application to (i) high resolution targets and (ii) uncluttered backgrounds. Thus, the project team concentrated its efforts in developing practical and robust waving detectors that can be applied to low resolution targets and arbitrary background clutter for outdoors environments. We address the robustness under different conditions by implementing two specialized waving detectors: (a) one suited for low resolution targets that relies on the local temporal consistency of optical flow-based features and (ii) the second one suited for arbitrary clutter on the image, which relies on data mining of a very large set of spatio-temporal features. In the following we describe the techniques utilized for each detector.

**Figure 23.**
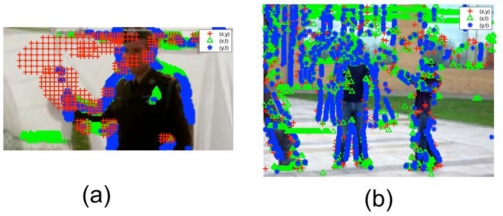
2D Harris corner detection on two frames.

1. Local temporal consistency of flow-based features [38]. This approach relies on a qualitative representation of body parts’ movements in order to build the model of waving patterns. Human activity is modeled using simple motion statistics information, not requiring the (time-consuming) pose reconstruction of parts of the human body. We use focus of attention (FOA) features [39], which compute optical flow statistics with respect to the target’s centroid. In order to detect waving activities at every frame, a boosting algorithm uses labeled samples of FOA features in a binary problem: waving vs not waving. We use the Temporal Gentleboost algorithm [40], which improves boosting performance by adding a new parameter to the weak classifier: the (short-term) temporal support of the features. We improve the noise robustness of the boosting classification by defining a waving event, which imposes the occurrence of a minimum number of single-frame waving classifications in a suitably defined temporal window.
2. Scale Invariant Mined Dense Corners Method. The generic human action detector [33] utilizes an over complete set of features, that are data mined to find the optimal subset to represent an action class.
  Space-time features have shown good performance for action recognition [41, 42]. They can provide a compact representation of interest points, with the ability to be invariant to some image transformations. While many are designed to be sparse in occurrence [40], we use dense simple 2D Harris corners [43]. While sparsity makes the problem tractable, it is not necessarily optimal in terms of class separability and classification.
  The features are detected in (x,y), (x,t) and (y,t) channels in the sequences at multiple image scales. This provides information on spatial and temporal image changes but is a far denser detection rate than 3D Harris corners [44] and encodes both spatial and spatio-temporal aspects of the data. The over complete set of features are then reduced through the levels of mining. Figure 23 shows the large amount of corners detected on two frames.

#### 8.2.7. Neighbourhood Grouping

**Figure 24.**
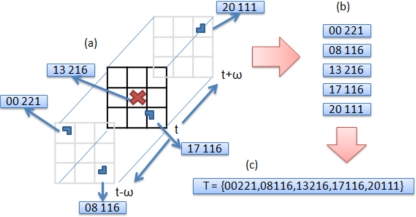
(a) Close-up example of a 3×3×3 neighborhood of an interest point, with size local features shown as corners. (b) Spatial and temporal encoding applied to each local feature. (c) Concatenating the local features into a transaction vector for this interest point.

Given a set of encoded corners, their relationships to other corners are used to build stronger compound features. Our approach is to define neighborhoods centered upon the feature that encode the relative displacement in terms of angle rather than distance hence achieving scale invariance. To do this, each detected interest point forms the centre of a neighborhood. The neighborhood is divided into 8 quadrants in the x, y, t domain which radiate from the centre of the neighborhood out to the borders of the image in *x*, *y* and one frame either side either *t* − 1 or *t*, *t* + 1 (see Figure 24b,c).

Each quadrant is given a label, all feature codes found within a unique quadrant are appended with the quadrant label. A vector of these elements is formed for every interest point found in the video sequence and contains the relative spatial encoding to all other features on the frame. This called a Transaction file, these are collected together into a large single file, containing around 2,000,000 transactions, where a single transaction contains around 400 items or encoded features. To condense or summarize this vast amount of information, a priori data mining is employed [45]. This computationally efficient approach, finds the frequent co-occurring configurations of encoded features among the tens of thousands of transaction vectors from the training sequences. Given a new query frame sequence, the features are detected and grouped as outlined above. This forms a new query set of transactions. A global classifier then exhaustively compares frequent mined configurations to the image feature groups in the query transaction set. It works as a voting scheme by accumulating the occurrences of the mined compound features. In order to learn the wave class for the URUS system, the training database is based on the KTH dataset [41]. We used the training setup proposed by the authors, in addition we added training examples from the URUS fixed cameras, and negative examples from other action classes and false positive detections from the URUS cameras.

#### 8.2.8. Fixed Camera Experiments

**Figure 25.**
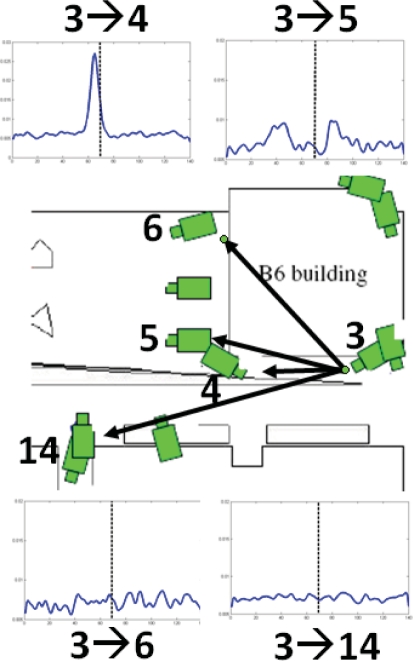
Inter camera temporal likelihoods.

A series of experiments were performed on the fixed camera system, to illustrate the incrementally learnt cross camera relations, the inter-camera relationships were learnt for a total of 5 days. The number of tracked objects on each camera was 200 per day. This is relatively low and made the region subdivision unsuitable after the second level of subdivision. Figure 25 shows resultant temporal likelihoods for a number of inter-camera links at a single subdivision level.

The black vertical line indicates a reappearance of zero seconds, it can be seen that there is strong links between cameras 3 and 4 and between 3 and 5, while there is no visible link between 3 and 6 and between 3 and 14. This is due to the increased distance and people will rarely reappear on cameras 6 and 14 after they were tracked on camera 3.

Table 1 shows the accuracy results of tracking people inter-camera. The inter-camera links were formed over up to 5 days and the test sequence consists of a 1 hour sequence on the cameras, with a total of 50 people tracked inter-camera. A 1 subdivision is a region per camera, 2 subdivision is the where any linked regions are subdivided. All people that moved inter-camera were ground-truthed and a true positive occurred when a person was assigned the same ID as that they were assigned on a previous camera.

**Figure 26.**
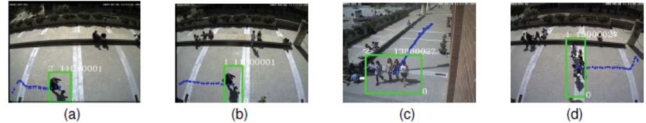
Cross camera tracking; (a) Person 11000001 on camera 11, (b) Person 11000001 correctly identified on camera 12 (c) Person 13000027 on camera 13 (d) Person 13000027 correctly identified on camera 12.

The column for 0 days indicates performance without learning the camera time and color relationships. It is poor generally due to large color variations inter-camera due to shadow and lighting changes. The 2 level subdivision initially performs poorly as it requires greater data to build relationships. However by 5 days significant improvement is shown for both one and two levels of region subdivision. Little performance is gained from the additional subdivision on this system due to the lower levels of traffic and low level of routes between the cameras due to their closeness. However for a more distributed system the additional detail of the region relationships would aid the tracking performance greater. Figure 26 gives example frames of tracking inter-camera for two separate people.

#### 8.2.9. Classification of Robots and Humans

**Figure 27.**
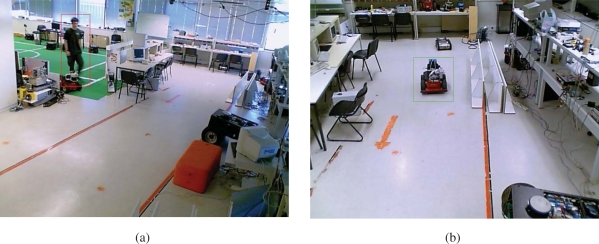
Examples of correct detections of (a) a person and (b) a Pioneer robot.

An initial test of the person *vs.* robot classifier was performed on the ISRobotNet. We grabbed five groups of sequences, where each one includes images from 10 cameras. One group with a person walking, another group with a different person walking, two groups with the same Pioneer robot moving in two different conditions, and the last group with a third person loitering. The people class videos have a total of 9,500 samples of the optical flow and the robot class videos have a total of 4,100 samples. We follow a cross validation approach to compare the classification result of the GentleBoost algorithm, so we build two different groups of training and testing sets. The best classification result for this setup was 95% recognition rate, provided by the MBH feature [35]. Figure 27 shows examples of correct detections of people and robots.

**Figure 28.**
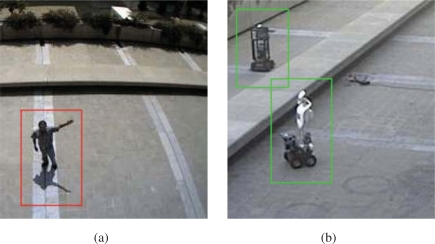
Examples of correct detections of (a) a person and (b) two robots.

From the results at the ISRobotNet, we select the MBH feature for a second group of tests, at the Barcelona Robot Lab. It is important to mention that the localization of some of the cameras on the Barcelona Robot Lab provide a point of view with large perspective effects on the optical flow features, so it is necessary to perform the training step with the new sequences. We grabbed sequences with people and robots performing naturally (*i.e.*, no given script or pre-defined actions known) for each camera. The actions include walking, loitering and waiting for person. The people class videos have a total of 19,444 samples of the optical flow and the robot class videos have a total of 2,955 samples. We follow the approach of the ISRobotNet test, building two different groups of training and testing sets. The average recognition rate of the two sets is 88.4%. Figure 28 shows examples of correct detections of people and robots.

#### 8.2.10. Gesture Detection with Local Temporal Consistency of Flow-based Features

The learning step of the Temporal Gentleboost algorithm with the FOA features was performed at the ISRobotNet. The training sequences have 4,229 frames (2,303 waving and 1,926 not waving), and the testing sequence has 4,600 frames (1,355 waving and 3,245 not waving). The FOA feature sampling is *π/*4. The support window of the Temporal boost algorithm is 20 frames. The event window size is 2 s (20 frames), considering a waving event if at least 60% of the single-frame classifications are positive. We follow a cross validation approach to compare the classification result of the GentleBoost algorithm, so we build two different groups of training and testing sets. The average performance of the two sets is 94.4%, on the ISRobotNet.

**Figure 29.**
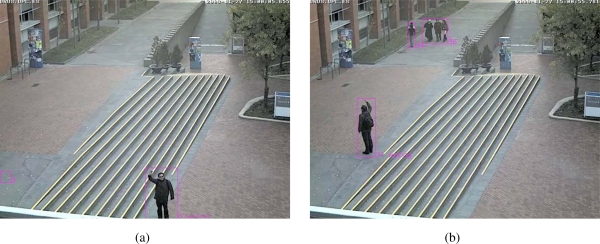
Examples of correct detections of a waving event, marked by the word waving on the bounding box.

We assume that the person is not translating on the image while performing the waving action, so the features selected by the Gentleboost algorithm on the ISRobotNet setup, are able to generalize well to different conditions, such as the target distance to the camera and the target size on the image. Thus, we use the parameters of the GentleBoost algorithm learned on the ISRobotNet in order to classify waving events in sequences grabbed at the Barcelona Robot Lab. The testing sequences have 3,288 (1,150 waving and 2,138 not waving). The recognition rate was 86.3%, which shows the generalization capabilities of the method. Figure 29 shows examples of the waving detection on a sequence recorded at the Barcelona Robot Lab.

It should be noted however that these performance results are preliminary, and a more exhaustive test campaign of the method is envisaged. It is necessary for instance, to validate the method under significant variations of illuminants (severe shadows, cloudy days, different hours of the day, *etc*.). This is the subject of further research.

### 8.3. Tracking with Mica2 Nodes

**Figure 30.**
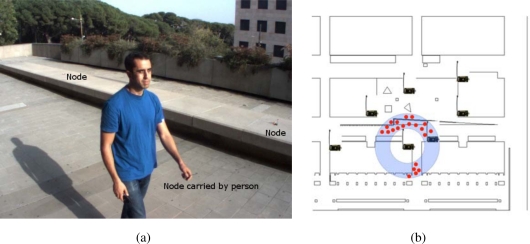
(a) Setup for person tracking based on radio-signal power. (b) Each power measurement delimits a feasible region within the map.

The WSN deployed in the environment can be used to estimate the position of mobile nodes of the network from the measured signal strength received by each static node, which is used for person tracking (see Figure 30a).

The algorithm to estimate the mobile node position is, as the robot localization algorithm discussed earlier, also based on particle filtering. In this case however, the current belief represents the position of the mobile node and is described by a set of particles; each particle representing a potential hypothesis about the actual position of the person carrying the node (see Figure 30a). At each filter iteration, kinematic models of the motion of the person and map information are used to predict the future position of the particles. The addition of the map information allows identifying and discarding unlikely motions with simple collision analysis. When the network of static nodes receives new messages from the mobile node, the weight of the different particles is updated with the RSSI indicator. By considering radio propagation models, it is possible to determine the likelihood of receiving a certain signal power as a function of the distance from the particle to the receiving node [46]. Each transmission restricts the position of the particles to an annular shaped area around the receiving node (see Figure 30b).

**Figure 31.**
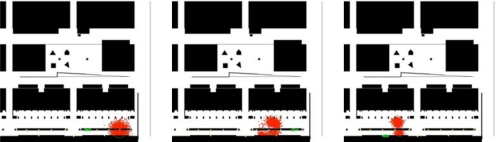
The evolution of the tracking estimates. Particle evolution (in red), and signal strength of each node (in green).

**Figure 32.**
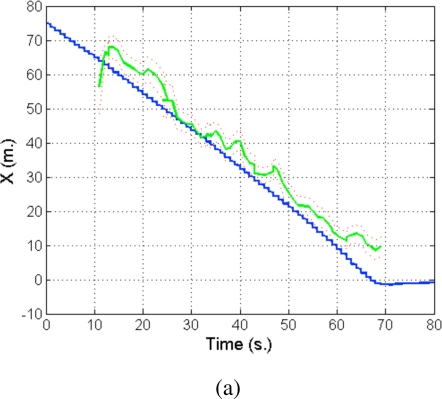
Estimated position of the person (green) and its standard deviation (red), compared with the position of the robot (blue) during a guiding mission.

As a result, the filter provides an estimate of the 3D position of the mobile node with one meter accuracy (depending on the density of nodes in the network). Figure 31 shows the evolution of the particles for a particular person guiding experiment at the Barcelona Robot Lab. Figure 32 shows the estimated position of the person estimated by the WSN, compared to that of the guiding robot which is in average about 3 meters ahead of the tracked person. The red dots indicate individual estimates of the particle filter and are shown to give an idea of the distribution of the localization estimate.

#### 

##### Decentralized Tracking with Cameras and Wireless Sensor Network

The full system can take advantage of all the information sources (cameras, robots, WSN) to improve the reliability and accuracy of the different services, as for example in tracking applications. In order to illustrate the benefits of the data fusion process described in Section 6, results from a simple setup are presented here. This setup consists of two fixed cameras and a WSN. The objective was to track one person cooperatively. In the experiment, the person is not always in the field of view of the cameras, appearing and disappearing from the image plane several times.

Three instances of the decentralized algorithm summarized in Section VI (see [26] for a thorough description) are launched, processing each camera’s data and the WSN estimations. They communicate to exchange estimated trajectories. The received data is fused with the local estimations (tracks on the image plane), leading to a decentralized tracking of the person.

**Figure 33.**
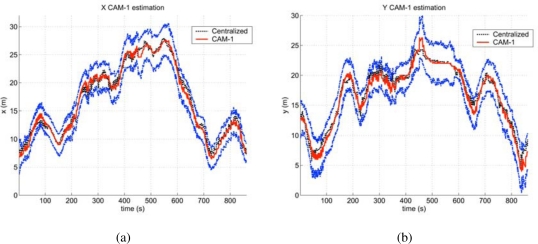
Estimated X and Y by the camera (red) compared to the centralized estimation (black), with the 3-sigma bound. The estimations are nearly the same, except during times when no communications occur.

Figure 33 shows the estimated person position (X and Y coordinates) compared with a centralized estimation (employing a central EKF filter). One important benefit from the system is that, if the communications channels are active, the different elements have nearly the same information. That way, one robot or camera, even not seeing the person, can know where it is. Also, the uncertainty is decreased due to the redundancies in the system.

## 9. Lessons Learned

Sensor fusion has proven to be a difficult undertaking in the URUS project. The issues that make it difficult include heterogeneity and latency of sensors and systems, the mobility of some of the sensors translating to network connectivity issues, and the challenges of environment perception in outdoor dynamic environments.

With regards to robot localization, we report here on results for a pair of experimental sessions that lasted for nearly three hours of autonomous navigation, and traversing over 3.5 km. The session included 35 navigation requests of which 77% of them were completed successfully. Of the 8 missions that did not reach completion, 1 was due to hardware malfunction of the Wifi interface, and 7 were due to software glitches. Of these, 5 resulted in poor localization, 1 was due to a failure in the path execution module, and 1 was the cause of limited obstacle avoidance capabilities.

The URUS localization module has room for improvement especially for those areas in the environment in which 3D perception is still an issue. This is, on ramps and near stairs. Moreover, path planning should also be improved to more robustly avoid obstacle-occluded intermediate waypoints. Furthermore, the robot sensing capabilities should also be enhanced. Our laser-based localization module cannot deal consistently with glass walls and in situations with extreme illumination conditions. Finally little but dangerous obstacles such as small holes and curbs in the path remain a perception challenge for Segway-type robotic platforms.

For the case of detection and tracking, the use of delayed states and a decentralized system was very important, as the switching between 3G and Wifi, which occurred from time to time in the system, produced significant latencies and communications drop-outs. The system was able to operate in guiding missions under these circumstances. For instance, when communications were re-established, the local estimates at each robot were combined with the accumulated information from other nodes, obtaining a good global estimate (see Section 8).

The point of fusing the data from cameras and the Mica2 WSN together was to increase reliability of the tracking compared to just using the cameras alone, as in cross camera tracking, occlusions and illumination changes will cause the tracking to fail between the uncalibrated non-overlapping cameras. A decentralized approach was used where each sensor had its own processing engine. This meant that they could work independently, and the system was not vulnerable to a sensor failing elsewhere in the system. This improves scalability and robustness of the system. A more detailed account of our decentralized tracking approach that fuses information from the camera network, the Mica sensors, and the onboard robot cameras is presented in [47].

## 10. Conclusions

We have presented in this article the architecture design of the URUS sensors for a Network Robot System. This architecture has been deployed in a real urban environment and is used to test and verify people assistant robot services. We have described the sensors used in several of the robots that we have built and the sensors that we have deployed in the urban environment. Moreover, we have described an example how to fuse such heterogeneous sensor information during urban robotic tasks using among other techniques, decentralized data fusion, information-based filtering, and asynchronous data handling.

The paper also discussed specific results on some of the application domains of the architecture. This paper presents significant advances in urban robotic sensor integration. During the course of our research, we have identified several important technological issues that must be solved in order to be able to unleash robots in cities at a commercial level. For instance, the WLAN communication systems and protocols are not appropriate for robot deployment, the laser range sensors must be redesigned to capture 3D data or the HRI sensors must be further developed.

Moreover we have realized that the experimental sites, such as the Barcelona Robot Lab, have allowed us to test the technology in real environments and in contact with people, a main feature required for city robots.

The URUS project is touching upon a very new and exciting technology, that of networked robotics services in urban outdoor environments. The choice of methods and architecture portrayed in this article is of course not the only choice, and perhaps not the most suited in some occasions, but we hope that with this articles and with our research endeavors, we can identify scientific and technological aspects for which the state of the art does not provide compelling solutions, paving the road for future work in the field.

## Figures and Tables

**Table 1.** Accuracy of fixed inter-camera tracking over days.

	Data amount (days)			

Method	0	1	2	5

1 Subdiv	34%	38%	56%	78%
2 Subdiv	34%	10%	60%	83%
